# Use of the linear regression method to evaluate population accuracy of predictions from non-linear models

**DOI:** 10.3389/fgene.2024.1380643

**Published:** 2024-05-31

**Authors:** Haipeng Yu, Rohan L. Fernando, Jack C. M. Dekkers

**Affiliations:** ^1^ Department of Animal Sciences, University of Florida, Gainesville, FL, United States; ^2^ Department of Animal Science, Iowa State University, Ames, IA, United States

**Keywords:** conditional mean, linear regression method, model adequacy, non-linear model, population accuracy

## Abstract

**Background:**

To address the limitations of commonly used cross-validation methods, the linear regression method (LR) was proposed to estimate population accuracy of predictions based on the implicit assumption that the fitted model is correct. This method also provides two statistics to determine the adequacy of the fitted model. The validity and behavior of the LR method have been provided and studied for linear predictions but not for nonlinear predictions. The objectives of this study were to 1) provide a mathematical proof for the validity of the LR method when predictions are based on conditional means, regardless of whether the predictions are linear or non-linear 2) investigate the ability of the LR method to detect whether the fitted model is adequate or inadequate, and 3) provide guidelines on how to appropriately partition the data into training and validation such that the LR method can identify an inadequate model.

**Results:**

We present a mathematical proof for the validity of the LR method to estimate population accuracy and to determine whether the fitted model is adequate or inadequate when the predictor is the conditional mean, which may be a non-linear function of the phenotype. Using three partitioning scenarios of simulated data, we show that the one of the LR statistics can detect an inadequate model only when the data are partitioned such that the values of relevant predictor variables differ between the training and validation sets. In contrast, we observed that the other LR statistic was able to detect an inadequate model for all three scenarios.

**Conclusion:**

The LR method has been proposed to address some limitations of the traditional approach of cross-validation in genetic evaluation. In this paper, we showed that the LR method is valid when the model is adequate and the conditional mean is the predictor, even when it is a non-linear function of the phenotype. We found one of the two LR statistics is superior because it was able to detect an inadequate model for all three partitioning scenarios (i.e., between animals, by age within animals, and between animals and by age) that were studied.

## Introduction

Advances in high-throughput genotyping have enabled the implementation of genomic prediction, which has facilitated the genetic improvement of animals and plants based on more accurate estimated breeding values (EBV) at an early age (e.g., Meuwissen et al., 2001; Dekkers and Hospital, 2002; Bernardo and Yu, 2007; Habier et al., 2011; Wolc et al., 2011; Morota et al., 2013). Various genomic prediction models have been proposed and prediction performance across or within models is usually evaluated by cross-validation (CV) methods (Utz et al., 2000; Meuwissen et al., 2001; Saatchi et al., 2011; Morota and Gianola, 2014). With CV, the data set is partitioned into training and validation sets, with the training set used to fit a prediction model and estimate the breeding values (BV) of individuals in the validation set. Prediction performance is commonly evaluated with the statistic of predictivity, which is the correlation coefficient between the EBV and phenotypes adjusted for fixed effects of individuals in the validation set. Scaling predictivity by the square root of heritability (*h* ^2^) provides an estimator for prediction accuracy of the EBV (Legarra et al., 2008; Serão et al., 2016), defined as the correlation between true and estimated breeding values. While accuracy estimated with CV has been widely used to quantify the performance of genomic prediction models, pre-correcting phenotypes in the validation set using estimates of fixed effects obtained using the whole data set will overestimate the accuracy when multiple levels of fixed effects are present (Legarra and Reverter, 2018). Additional limitations include that it cannot be applied to complex models (e.g., models with random regression, sex limited traits, maternal effects, additive and non-additive effects), indirect traits (e.g., unobserved latent traits), and traits with low *h* ^2^ (Legarra and Reverter, 2018).

To address these limitations of the CV methodology, Legarra and Reverter (2018) proposed a linear regression (LR) method to estimate the accuracy of genomic prediction implicitly assuming the fitted model is correct. The LR method quantifies the population accuracy of predictions based on the correlation between EBV of individuals in the validation set estimated using the training set and the EBV of those same individuals estimated using the combined training and validation sets. In the LR method literature, the training set is referred to as the partial data set (*p*) and the combined training and validation data set is referred to as the whole data set (*w*). The LR estimator of population accuracy is theoretically justified only when the fitted model is adequate. Thus the LR method also provides two statistics that can be used to check if the model is adequate.

The LR method was mathematically justified by Legarra and Reverter (2018) based on results from Reverter et al. (1994). Macedo et al. (2020) investigated the behavior and properties of the LR method by analyzing simulated data with pedigree-based genetic models. They studied the LR estimators of population bias and accuracy of predictions by using wrong values of *h* ^2^ in the analysis and by fitting wrong models that ignored the environmental trend through the simulated generations, and claimed that “the LR method works reasonably well for detection of bias when the model used is robust or close to the true model, and that it works well for estimation of accuracy even when the model is not good.” Validity and performance of the LR method for a non-linear model was explored by Bermann et al. (2021). In their study, they evaluated the performance of the LR method by fitting a threshold model to simulated data and also applied it to real data to estimate the increase in accuracy by adding genomic information. Based on the results from the simulated data, they concluded that the LR method can be useful to estimate the directions of bias, dispersion, and accuracy, though the LR estimators had different magnitudes compared to the true estimators. The original proof of the LR method (Legarra and Reverter, 2018) was based on the setting where the whole data set had additional phenotype records relative to the partial data set. Belay et al. (2022) recently showed that the LR method can also be applied to the setting where the whole data set has additional genotypes (rather than phenotypes) relative to the partial data set. They used the LR method to evaluate the bias and accuracy in single-step genomic predictions.

While the validity and performance of LR method has been explored using linear and non-linear models in previous studies (Macedo et al., 2020; Bermann et al., 2021), a mathematical proof of its validity for non-linear methods of prediction has not yet been presented. In addition, studies about the performance of the LR method when a model other than the true model is fitted are still relatively scarce in the literature. The objectives of this study were to 1) present a mathematical proof of the validity of the LR method when predictions are based on the conditional mean, regardless of whether it is a linear or non-linear function of the data 2) investigate the ability of the LR method to detect whether the fitted model is adequate or inadequate, and 3) provide guidelines on how to partition the data set such that the LR method can detect the use of an inadequate model.

## Theory

### Notation

In the LR method, Legarra and Reverter (2018) used var(**x**) to denote the variance of a random element, *x*, sampled from a single realization of the random vector **x**, and Var(**x**) to denote the variance-covariance matrix for the vector **x**. Similarly, they used cov(**x**, **y**) to denote the covariance between randomly sampled *x*_*i*_ and *y*_*i*_ and Cov(**x**, **y**) to denote the covariance matrix between the vectors **x** and **y**. Let **u** denote the vector of BV of the validation animals, and ${\hat{u}}_{p}$ and ${\hat{u}}_{w}$ denote the vector of EBV of **u** obtained from partial data and whole data, respectively.

### Proof of the validity of the LR method when the conditional mean is used for prediction

Legarra and Reverter (2018) developed the LR method for best linear unbiased prediction (BLUP) by showing that $Cov\left({\hat{u}}_{w},{\hat{u}}_{p}\right)=Var\left({\hat{u}}_{p}\right)$ , using the results from Reverter et al. (1994) and the result that $Cov\left(u,{\hat{u}}_{p}\right)=Var\left({\hat{u}}_{p}\right)$ and $E\left({\hat{u}}_{p}\right)=E\left({\hat{u}}_{w}\right)=E\left(u\right)$ from Henderson (1982). Using the above results for BLUP, they showed that $E\left[cov\left({\hat{u}}_{w},{\hat{u}}_{p}\right)\right]=E\left[var\left({\hat{u}}_{p}\right)\right]=E\left[cov\left(u,{\hat{u}}_{p}\right)\right]$ , and thus justified that $cov\left(u,{\hat{u}}_{p}\right)$ can be quantified by computing $cov\left({\hat{u}}_{w},{\hat{u}}_{p}\right)$ .

It is well known that whenever ${\hat{u}}_{p}$ is the conditional mean of the unobservable random variable **u**_*p*_ given observed phenotype, $Cov\left(u,{\hat{u}}_{p}\right)=Var\left({\hat{u}}_{p}\right)$ (Rao, 1973). Further, based on the double expectation theorem (DeGroot and Schervish, 2002), the condition mean is known to be unbiased in the sense that $E\left(\hat{u}\right)=E\left(u\right)$ . Thus it follows that, if the conditional mean is used for prediction, $E\left({\hat{u}}_{p}\right)=E\left({\hat{u}}_{w}\right)=E\left(u\right)$ . Below, we show that whenever ${\hat{u}}_{p}$ is based on conditional means, $Cov\left({\hat{u}}_{w},{\hat{u}}_{p}\right)=Var\left({\hat{u}}_{p}\right)$ . Then, using the results from Legarra and Reverter (2018), it follows that $E\left[cov\left({\hat{u}}_{w},{\hat{u}}_{p}\right)\right]=E\left[var\left({\hat{u}}_{p}\right)\right]=E\left[cov\left(u,{\hat{u}}_{p}\right)\right]$ and $cov\left(u,{\hat{u}}_{p}\right)$ = $cov\left({\hat{u}}_{w},{\hat{u}}_{p}\right)$ for predictions using conditional means, regardless of whether the conditional mean is a linear or non-linear function of the data.

Here we show that $Cov\left({\hat{u}}_{w},{\hat{u}}_{p}\right)=Var\left({\hat{u}}_{p}\right)$ when predictions are based on conditional means, which may be non-linear. Let

$$y_{w}=\left[\begin{array}{c} y_{p}\\ y_{r} \end{array}\right],$$

where **y**_*w*_, **y**_*p*_, and **y**_*r*_ indicate vectors of phenotype records in the whole, partial, and validation (remaining) data set, respectively. In the Supplementary Appendix, we show $E_{y_{r}|y_{p}}\left({\hat{u}}_{w}|y_{p}\right)={\hat{u}}_{p}$ , which represents the expectation over the conditional distribution of **y**_*r*_ given **y**_*p*_ and will be used in the following proof.

Now, we write the $Cov\left({\hat{u}}_{w},{\hat{u}}_{p}\right)$ as:

$$\begin{array}{ll} Cov\left({\hat{u}}_{w},{\hat{u}}_{p}\right) & =E_{y_{w}}\left[\left({\hat{u}}_{w}-\theta_{w}\right){\left({\hat{u}}_{p}-\theta_{p}\right)}^{\prime }\right]\\ & =E_{y_{w}}\left[{\hat{u}}_{w}{\left({\hat{u}}_{p}-\theta_{p}\right)}^{\prime }\right]-E_{y_{w}}\left[\theta_{w}{\left({\hat{u}}_{p}-\theta_{p}\right)}^{\prime }\right]\\ & =E_{y_{w}}\left[{\hat{u}}_{w}{\left({\hat{u}}_{p}-\theta_{p}\right)}^{\prime }\right]-\theta_{w}E_{y_{w}}\left[{\left({\hat{u}}_{p}-\theta_{p}\right)}^{\prime }\right]\\ & =E_{y_{w}}\left[{\hat{u}}_{w}{\left({\hat{u}}_{p}-\theta_{p}\right)}^{\prime }\right]-\theta_{w}0^{\prime }\\ & =E_{y_{w}}\left[{\hat{u}}_{w}{\left({\hat{u}}_{p}-\theta_{p}\right)}^{\prime }\right]\\ & =E_{y_{w}}\left[\left({\hat{u}}_{w}-{\hat{u}}_{p}+{\hat{u}}_{p}\right){\left({\hat{u}}_{p}-\theta_{p}\right)}^{\prime }\right]\\ & =E_{y_{w}}\left[\left({\hat{u}}_{w}-{\hat{u}}_{p}\right){\left({\hat{u}}_{p}-\theta_{p}\right)}^{\prime }+{\hat{u}}_{p}{\left({\hat{u}}_{p}-\theta_{p}\right)}^{\prime }\right], \end{array}$$

where ***θ***_*p*_ and ***θ***_*w*_ are the expected values of ${\hat{u}}_{p}$ and ${\hat{u}}_{w}$ , respectively. The first term of this expectation can be shown to be null:

(see the Supplementary Appendix for derivation)

$$\begin{array}{ll} E_{y_{w}}\left[\left({\hat{u}}_{w}-{\hat{u}}_{p}\right){\left({\hat{u}}_{p}-\theta_{p}\right)}^{\prime }\right] & =E_{y_{p}}\left\{E_{y_{r}|y_{p}}\left[\left({\hat{u}}_{w}-{\hat{u}}_{p}\right){\left({\hat{u}}_{p}-\theta_{p}\right)}^{\prime }|y_{p}\right]\right\}\\ & =E_{y_{p}}\left\{E_{y_{r}|y_{p}}\left[\left({\hat{u}}_{w}-{\hat{u}}_{p}\right)|y_{p}\right]{\left({\hat{u}}_{p}-\theta_{p}\right)}^{\prime }\right\}\\ & =E_{y_{p}}\left[\left({\hat{u}}_{p}-{\hat{u}}_{p}\right){\left({\hat{u}}_{p}-\theta_{p}\right)}^{\prime }\right]=0, \end{array}$$

because, as shown in the Appendix that $E_{y_{r}|y_{p}}\left({\hat{u}}_{w}|y_{p}\right)={\hat{u}}_{p}$ . Thus, the $Cov\left({\hat{u}}_{w},{\hat{u}}_{p}\right)$ becomes:

$$\begin{array}{ll} Cov\left({\hat{u}}_{w},{\hat{u}}_{p}\right) & =E_{y_{w}}\left[{\hat{u}}_{p}{\left({\hat{u}}_{p}-\theta_{p}\right)}^{\prime }\right]\\ & =E_{y_{p}}\left[{\hat{u}}_{p}{\left({\hat{u}}_{p}-\theta_{p}\right)}^{\prime }\right]\\ & =Var\left({\hat{u}}_{p}\right). \end{array}$$

The proof of $Cov\left({\hat{u}}_{w},{\hat{u}}_{p}\right)=Var\left({\hat{u}}_{p}\right)$ shows the LR method also holds for non-linear predictions. This proof is similar in principle to that provided by Belay et al. (2022), but we recognize that it is not limited to BLUP, as invoked in that study, but is applicable to any method of prediction based on the conditional mean (Fernando and Gianola, 1986), including for non-linear models.

## Data simulation

A longitudinal data set of body weights in pigs was simulated with 20 replicates to evaluate the behavior of the LR method for non-linear models. Body weights of 1,500 individuals in the same generation from 70 to 500 days of age were simulated using a combination of multi-trait QTL effects that were randomly sampled from a multivariate normal distribution and a Gompertz growth model. Only 30 bi-allelic independent QTL were simulated to make the computations manageable. Following Brossard et al. (2009), the body weight of individual *i* at age *t* (*BW*_*it*_) was simulated as:

$$BW_{it}=\text{g}\left(t;\theta_{i}\right)+\epsilon_{it},$$ (1)

where $\theta_{i}=\left[\begin{array}{ccc} Age115_{i} & Shape_{i} & BW65_{i} \end{array}\right]$ refers to three underlying latent variables for pig *i* of age at 115 kg, a shape parameter, and body weight at 65 days, and *ϵ*_*it*_ is the residual. We simulated heterogeneous residuals to mimic the real growth data for pigs using three different residuals across days 70–500 (i.e., 70–167 days: $\sigma_{\epsilon_{1}}^{2}=3.0$ , 168–334 days: $\sigma_{\epsilon_{2}}^{2}=4.0$ , and 335–500 days: $\sigma_{\epsilon_{3}}^{2}=8.0$ ) based on estimates obtained from actual body weight data by Yu et al. (2022). In Eq. 1, g(.) indicates a reparameterized Gompertz function, which replaces the initial and mature *BW*_*i*_ with *BW*65_*i*_ and *Age*115_*i*_, respectively (Brossard et al., 2009):

$$\text{g}\left(t;\theta_{i}\right)=115\times {\left(\frac{115}{BW65_{i}}\right)}^{\left(-\frac{e^{\left(-Shape_{i}\left(Age115_{i}-65\right)\right)}-e^{\left(-Shape_{i}\left(-65+t\right)\right)}}{-1+e^{\left(-Shape_{i}\left(Age115_{i}-65\right)\right)}}\right)}.$$

The three underlying latent variables ***θ***_*i*_ for individual *i* were considered correlated and modeled with a multivariate QTL effects model.

$$\theta_{i}=\mu +\overset{p}{\underset{j=1}{\sum }}m_{ij}\alpha_{j}+e_{i},$$

where ***μ*** is a 3 × 1 vector with the intercepts for each latent variable, *m*_*ij*_ is the genotype covariate (0, 1, 2) of individual *i* at the *jth* QTL, ***α***_*j*_ is a vector of random substitution effects for the three latent variables for the *j*th QTL, and ***e***_***i***_ is a vector of random environmental effects associated with each latent variable. Based on the results of Yu et al. (2022), the variance-covariance matrix used for simulation of random environmental effects (**Σ**_*e*_) was equal to $\left[\begin{array}{ccc} 3.65\times 10^{-3} & 1.05\times 10^{-3} & 5.51\times 10^{-3}\\ 1.05\times 10^{-3} & 3.52\times 10^{-2} & -1.44\times 10^{-2}\\ -5.51\times 10^{-3} & -1.44\times 10^{-2} & 3.05\times 10^{-2} \end{array}\right]$ . The variance-covariance used to simulate QTL effects for the three latent variables (**Σ**_*α*_) was arbitrarily but without loss of generality derived by dividing the environmental variance-covariance by the number of QTL (i.e., 30).

## Data analysis models

Using the QTL as markers, without loss of generality, the simulated data were analyzed to examine the ability of the LR method to determine whether the fitted model is adequate or inadequate by fitting two Bayesian hierarchical models: 1) the Gompertz model that was used for simulation, i.e., the true model, and 2) a quadratic growth model, i.e., a wrong model. The prediction performances of these two models were evaluated using the LR method across 20 replicates. To reduce the required number of replicates, the variance components that were used to simulate the data were fitted into the true and wrong models for analysis. All analyses were performed in Julia (Bezanson et al., 2017).

While Bayesian hierarchical inference with pedigree information has been used in previous studies to integrate growth models into genetic evaluations (Varona et al., 1999; Cai et al., 2012), we used the Bayesian hierarchical Gompertz growth model (BHGGM) developed by Yu et al. (2022), which integrates a Gompertz growth model, i.e., the true model, with a multi-trait marker effects models. Following Eq. 1, the three underlying latent variables in the Gompertz growth model were assigned the following prior:

$$\theta_{i}∼MVN\left(\mu +\overset{p}{\underset{j=1}{\sum }}m_{ij}\alpha_{j},\Sigma_{e}\right),$$

where **Σ**_*e*_ is the environmental variance-covariance matrix defined above in the simulation. The prior for *ϵ*_*it*_ had a null mean and age specific variances (as described above in Eq. 1) to allow fitting heterogeneous residuals. Flat priors were assigned to ***μ*** and the prior for ***α***_*j*_ followed $MVN\left(0,\Sigma_{\alpha }\right)$ , where **Σ**_*α*_ is the marker variance-covariance matrix used for simulation. All parameters followed the same dimension as defined in the section of data simulation.

For analysis using the Bayesian hierarchical quadratic growth model (BHQGM), i.e., the wrong model, the following quadratic growth model was fitted:

$$BW_{it}=\text{f}\left(t;\theta_{Qi}\right)+\epsilon_{it},$$

where f (.) is a quadratic function:

$$\text{f}\left(t;\theta_{Qi}\right)=b_{i0}+b_{i1}t+b_{i2}t^{2}.$$

the parameter $\theta_{Qi}=\left[\begin{array}{ccc} b_{i0} & b_{i1} & b_{i2} \end{array}\right]$ refers to three underlying latent variables for individual *i* and were assigned the same multivariate normal prior as ***θ***_***i***_ in BHGGM. The other parameters of the BHQGM also used the same priors as the corresponding parameters of the BHGGM.

### Design of the partial and validation data sets

**FIGURE 1:**
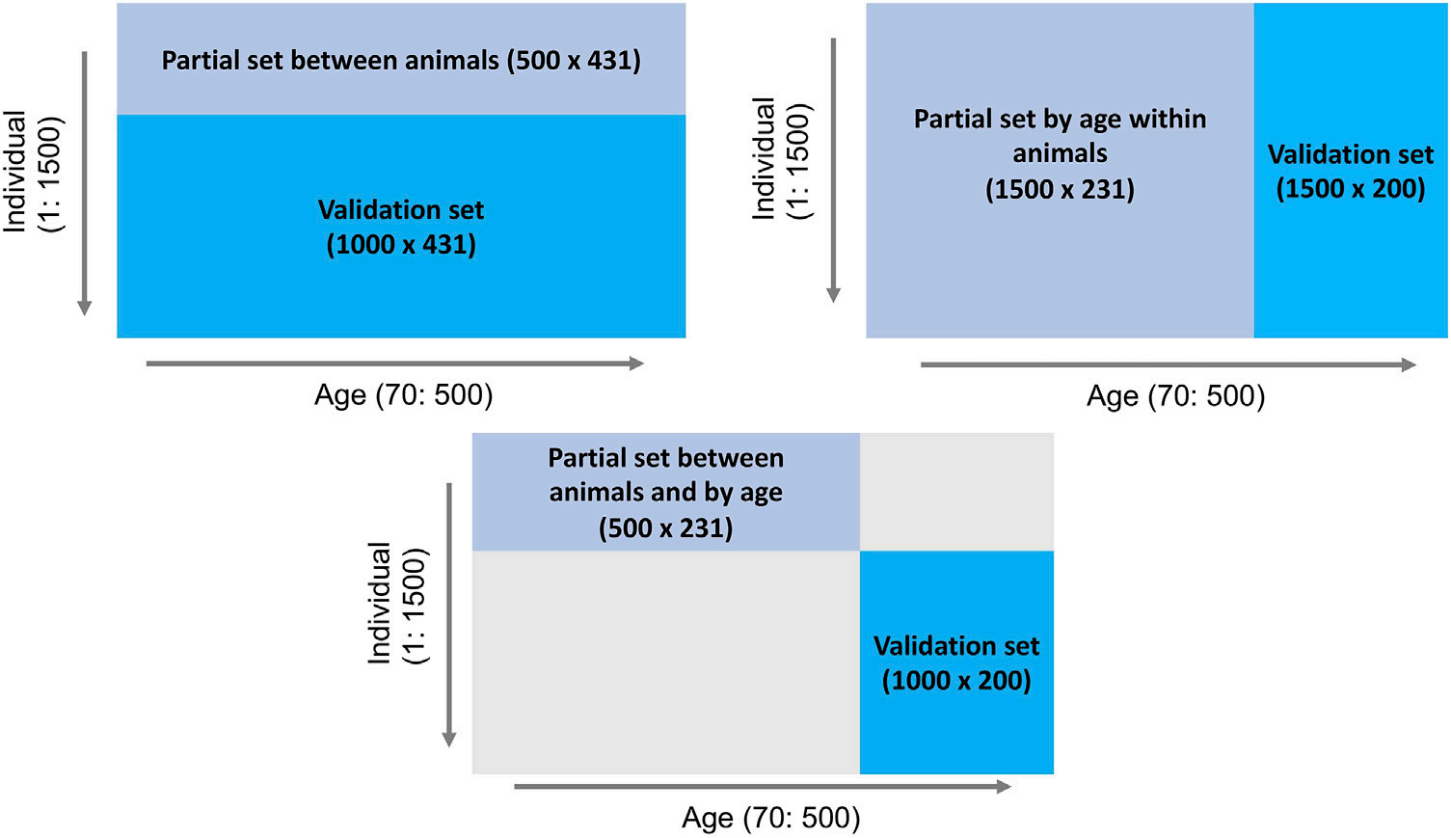
Outline of three data partitioning scenarios to create partial and validation sets for the LR method.

To investigate the behavior of the LR method, three partitioning scenarios (Figure 1) were implemented: 1) between animals: phenotype records for days 70–500 of the first 500 individuals comprised the partial set and the phenotypes of the remaining 1,000 individuals were assigned to the validation set, 2) by age within animals: phenotypes for days 70–300 of all 1,500 individuals comprised the partial set and all 1,500 individuals and their phenotypes from days 301–500 were considered as the validation set, and 3) between animals and by age: phenotypes for days 70–300 of the first 500 individuals comprised the partial set and phenotypes for days 301–500 for the remaining 1,000 individuals were assigned to the validation set. The EBV of interest were those for body weight for individuals in the validation set across days, predicted based on the partial set for each of the three scenarios.

### LR method

As described below, following Legarra and Reverter (2018), two statistics are used to check whether the model is adequate.

1. The first statistic is $\hat{\Delta }\;=\bar{{\hat{u}}_{p}}-\bar{{\hat{u}}_{w}}$ , which is the difference between the mean EBV of individuals in the validation set estimated based on the partial and whole data sets, respectively. This is an estimator of $\Delta \;=\bar{{\hat{u}}_{p}}-\bar{u}$ . A deviation of $\hat{\Delta }$ from 0 indicates the model is not adequate.
2. The second statistic is obtained by regressing ${\hat{u}}_{w}$ on ${\hat{u}}_{p}$ , ${\hat{b}}_{wp}\;=\frac{cov\left({\hat{u}}_{w},{\hat{u}}_{p}\right)}{var\left({\hat{u}}_{p}\right)}$ , which is an estimator of the regression of *u* on ${\hat{u}}_{p}$ : $b_{up}=\frac{cov\left(u,{\hat{u}}_{p}\right)}{var\left({\hat{u}}_{p}\right)}$ . A deviation of ${\hat{b}}_{wp}$ from 1 indicates the model is not adequate.

This estimator was referred to as an estimator of dispersion by Legarra and Reverter (2018) and of inflation by Belay et al. (2022), where 1 indicates no bias. Suppose animals are selected based on EBV to increase the values of a trait. Then if the true regression coefficient is less than 1, the BV of selected candidates is expected to be lower than their EBV, which indicates an upward bias of the EBV of the selected animals. On the other hand, when ${\hat{b}}_{wp}$ is larger than 1, the BV of selected candidates is expected to be higher than their EBV, which indicates a downward bias of the EBV of the selected animals.

Provided the model is adequate, in the LR method, population accuracy is estimated as ${\hat{\rho }}_{p}\;=\frac{cov\left({\hat{u}}_{w},{\hat{u}}_{p}\right)}{\sqrt{\hat{var\left(u\right)}\times var\left({\hat{u}}_{p}\right)}}$ , where $\hat{var\left(u\right)}$ refers to an estimate of the genetic variance of individuals in the validation set. This estimate was obtained by Gibbs sampling as: $\hat{var\left(u\right)}=\frac{1}{n_{\mathrm{trn}}}\sum_{j=1}^{n_{\mathrm{trn}}}u_{j}^{2}-{\left(\frac{1}{n_{\mathrm{trn}}}\sum_{j=1}^{n_{\mathrm{trn}}}u_{j}\right)}^{2}$ , where *u*_*j*_ refers to a sample of the BV of individual *j* from its posterior distribution in the training set and *n*_*trn*_ is the total number of individuals in the training set (Fernando et al., 2017).

In addition to $\hat{\Delta }$ , ${\hat{b}}_{wp}$ , and ${\hat{\rho }}_{p}$ , we also calculated the Δ, *b*_*wp*_, and *ρ*_*p*_, which are the “true” values of these quantities by using the simulated values of **u** in place of ${\hat{u}}_{w}$ in the formulas. Note that these “true” values can only be computed in a simulation study, and they are used here to study the performance of the LR method.

The means of Δ and $\hat{\Delta }$ were calculated for each day of age across all animals in the validation set. These mean values were averaged across days within each replicate to test whether their mean was significantly different from 0 using a t-test. Similarly, true and estimated regression coefficients were averaged across days within each replicate to test whether their mean was significantly different from 1 using a t-test. Additionally, we tested the difference between $cov\left({\hat{u}}_{w},{\hat{u}}_{p}\right)$ and $cov\left(u,{\hat{u}}_{p}\right)$ by first calculating the mean difference across age for each of 20 replications and then used a t-test to determine if these differences were significantly different from zero.

## Results

**FIGURE 2:**
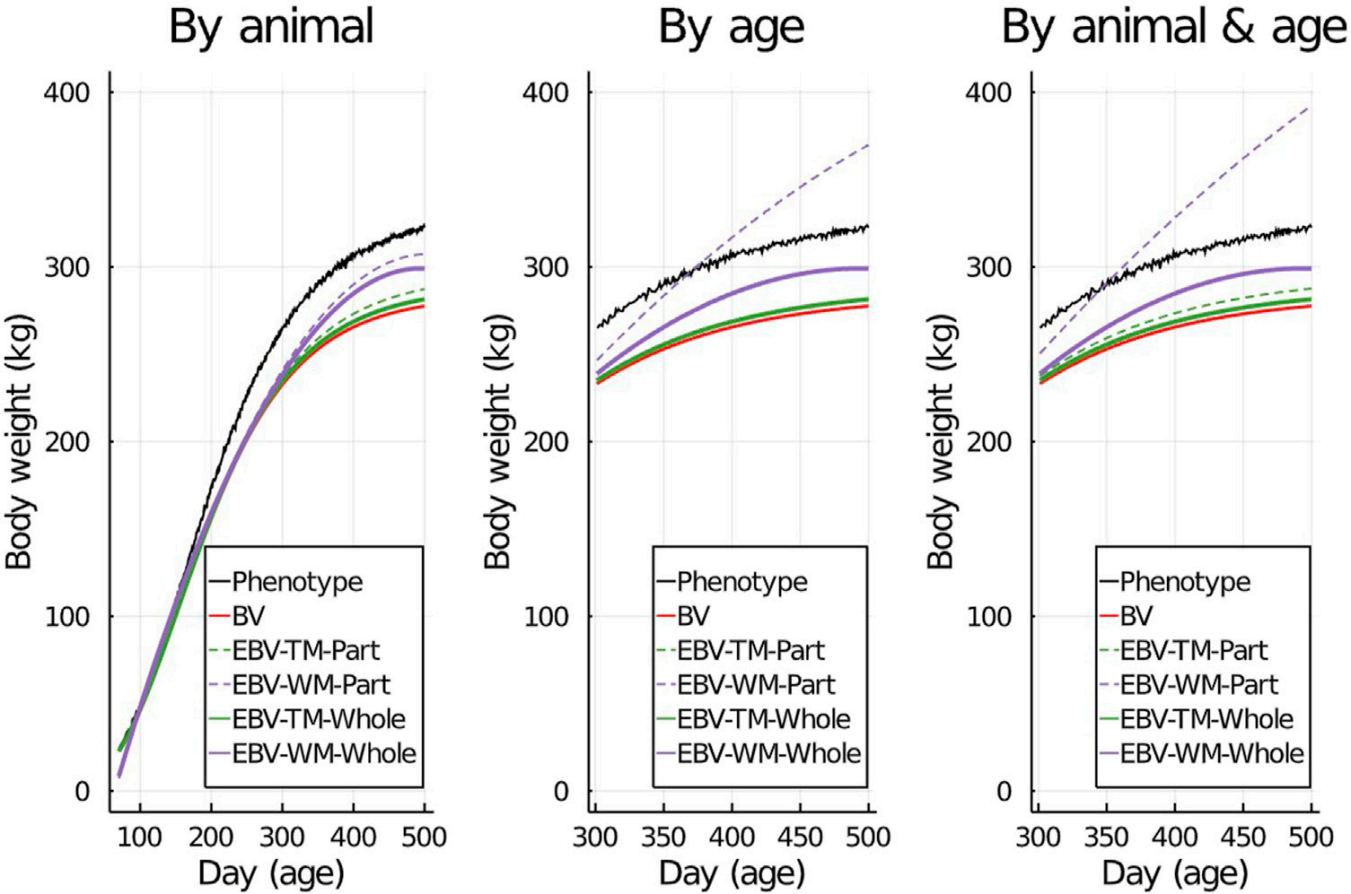
An example showing one randomly selected individual’s simulated phenotypes, true breeding values (BV), and estimated breeding values (EBV) for body weight by age using the true model (TM) and the wrong model (WM) when the data set was partitioned using different scenarios.

To visualize the prediction performances across the fitted models and partitioning scenarios, we randomly picked one individual from the validation set and displayed its simulated data against its predictions in Figure 2. Both simulated body weight phenotypes, true BV, and EBV of the selected individual were displayed. The predicted data included the EBV based on the partial and the whole data sets for the three partitioning scenarios (Figure 2).

### Evaluating model adequacy

**FIGURE 3:**
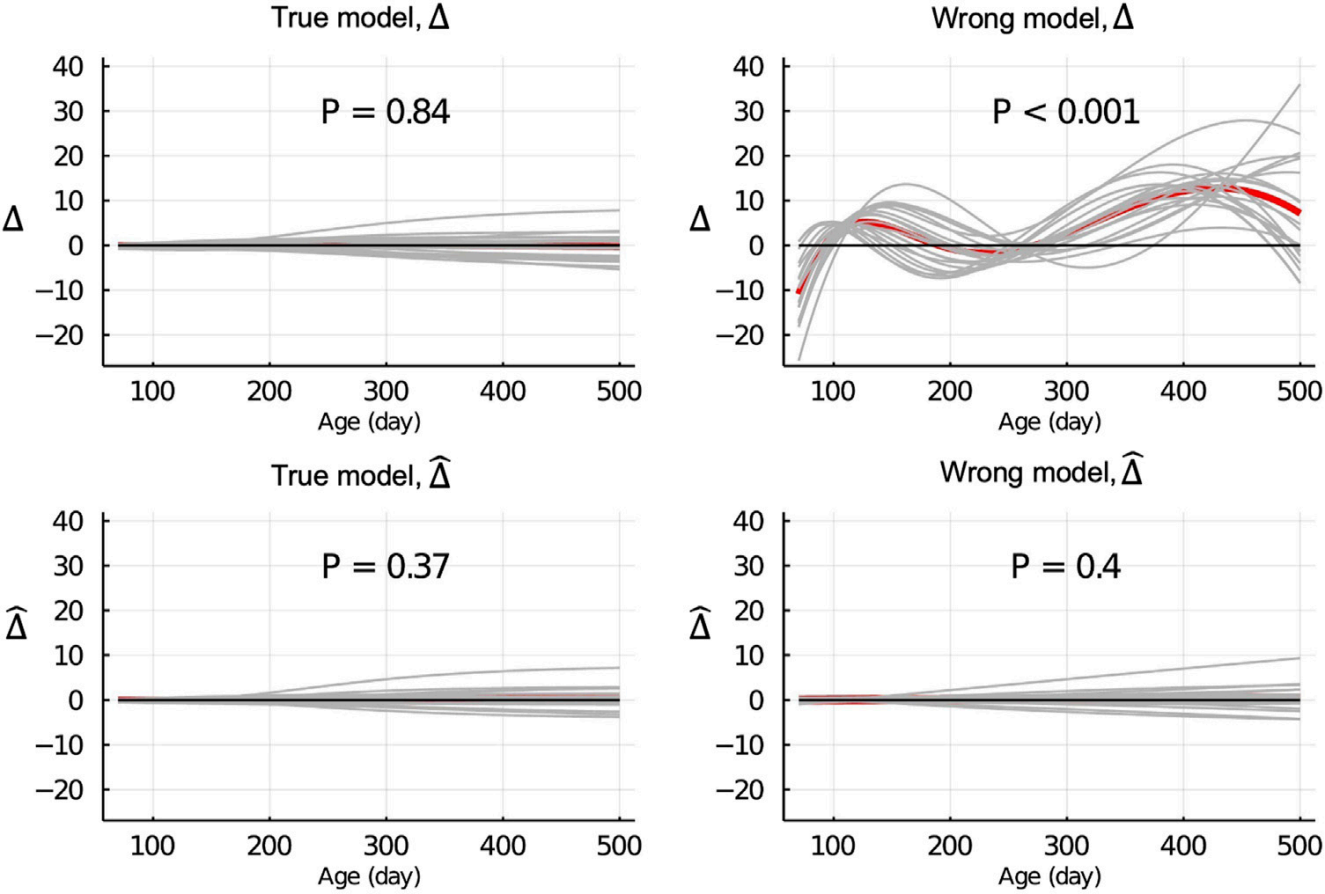
$\Delta =\bar{{\hat{u}}_{p}}-\bar{u}$ and $\hat{\Delta }=\bar{{\hat{u}}_{p}}-\bar{{\hat{u}}_{w}}$ of EBV of body weights for each day of age when the true or wrong model was fitted and when partitioning the data between animals. Grey lines are results of 20 simulation replicates, the red line is the mean of 20 replicates, and the black line indicates bias = 0. P refers to significance of tests for the difference between Δ or $\hat{\Delta }$ and 0.

Figure 3 shows the Δ and $\hat{\Delta }$ for EBV of body weight for each day when the data were partitioned between animals. When the true model (BHGGM) was used, both the Δ and $\hat{\Delta }$ were symmetrically distributed around 0 for each day, and their mean was not significantly different from 0 (*p* = 0.84 and = 0.37, respectively). In contrast, when the wrong model (BHQGM) was used, the mean of the Δ was significantly different from 0 (*p* < 0.001), but the $\hat{\Delta }$ were symmetrically distributed around 0 for each day and their mean was not significantly different from 0 (*p* = 0.4).

**FIGURE 4:**
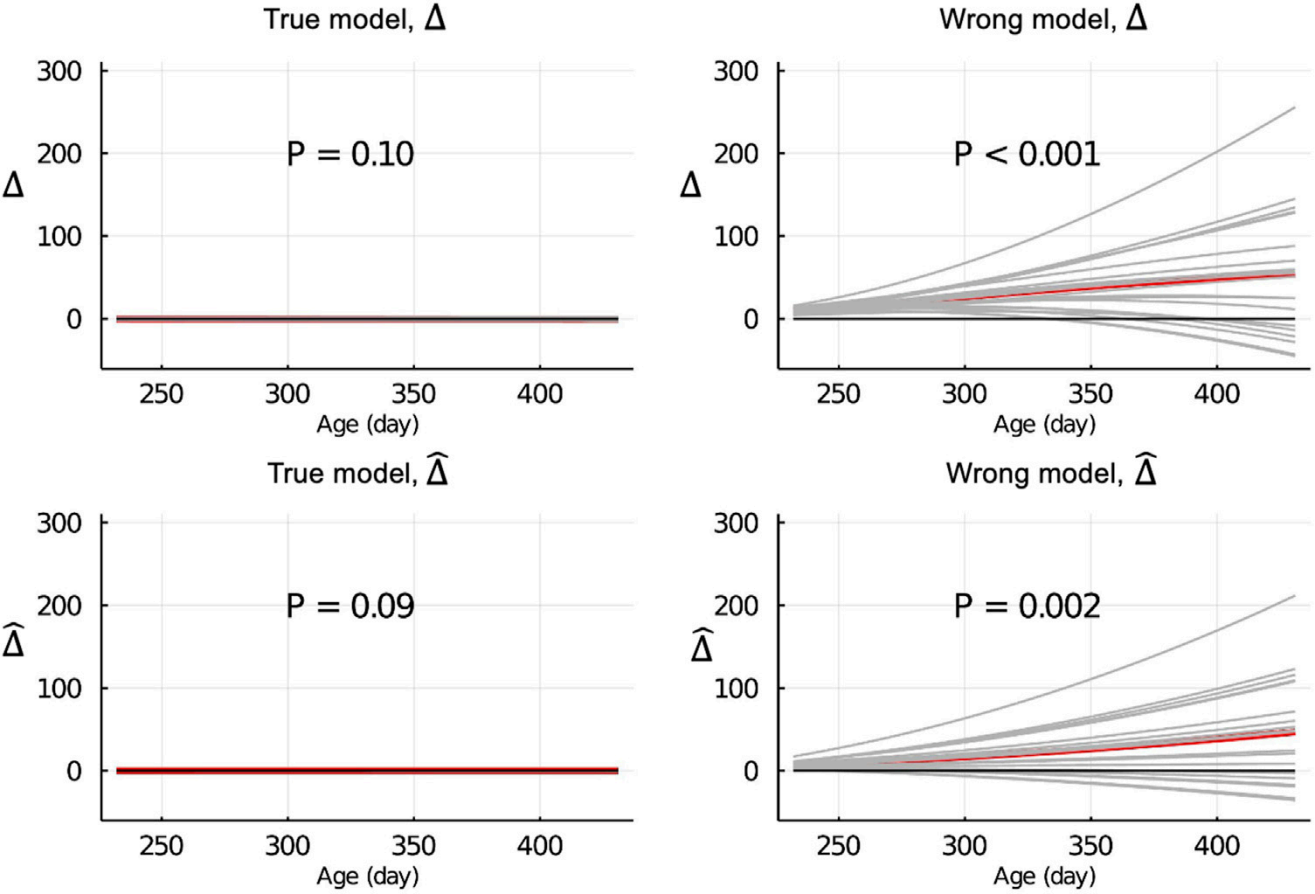
$\Delta =\bar{{\hat{u}}_{p}}-\bar{u}$ and $\hat{\Delta }=\bar{{\hat{u}}_{p}}-\bar{{\hat{u}}_{w}}$ of EBV of body weights for each day of age when the true or wrong model was fitted and when partitioning the data by age within animals. Grey lines are results of 20 simulation replicates, the red line is the mean of 20 replicates, and the black line indicates bias = 0. P refers to significance of tests for the difference between Δ or $\hat{\Delta }$ and 0.

Figure 4 shows the Δ and $\hat{\Delta }$ for EBV of body weights for each day when the data were partitioned by age within animals. When the true model was used, both the Δ and $\hat{\Delta }$ were symmetrically distributed around 0 for each day, and their mean was not significantly different from 0 (*p* = 0.10 and = 0.09, respectively). When the wrong model was used, the Δ and $\hat{\Delta }$ were significantly different from 0 (*p* < 0.001 and = 0.002, respectively). Results for the partitioning between animals and by age were consistent with those in Figure 4 and are shown in Supplementary Figure S1.

**FIGURE 5:**
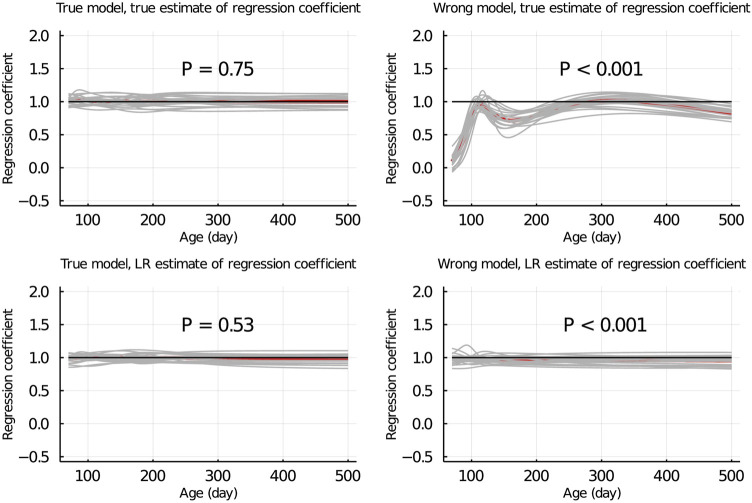
True and LR estimates of regression coefficient of EBV of body weights for each day of age when the true or wrong model was fitted and when partitioning the data between animals. The true and LR estimates of regression coefficient are defined by regressing *u* on ${\hat{u}}_{p}$ , and ${\hat{u}}_{w}$ on ${\hat{u}}_{p}$ , respectively. Grey lines are results of 20 simulation replicates, the red line is the mean of 20 replicates, and the black line indicates regression coefficient = 1. P refers to significance of tests for the difference between true or LR estimate of regression coefficient and 1.

**FIGURE 6:**
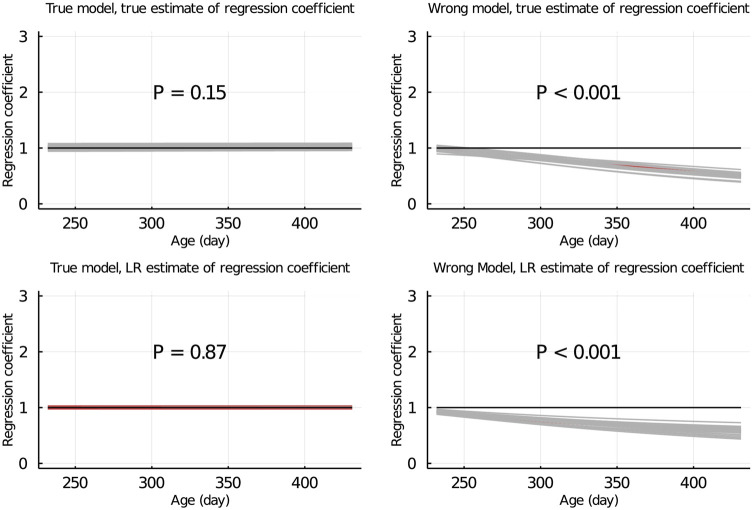
True and LR estimates of regression coefficient of EBV of body weights for each day of age when the true or wrong model was fitted and when partitioning the data by age within animals. The true and LR estimates of regression coefficient are defined by regressing *u* on ${\hat{u}}_{p}$ , and ${\hat{u}}_{w}$ on ${\hat{u}}_{p}$ , respectively. Grey lines are results of 20 simulation replicates, the red line is the mean of 20 replicates, and the black line indicates regression coefficient = 1. P refers to significance of tests for the difference between true or LR estimate of regression coefficient and 1.

Figure 5 shows the true and LR estimates of the regression coefficient of EBV for body weights for each day when the data were partitioned between animals. When the true model was used, both the true and estimated regression coefficients were symmetrically distributed around 1 for each day, and their mean was not significantly different from 1 (*p* = 0.75 and = 0.53, respectively). When the wrong model was used, the true and LR estimates of the regression coefficient were significantly different from 1 (*p* < 0.001). Results for the partitioning by age within animals and between animals and by age were consistent with those in Figure 5 and are given in Figure 6; Supplementary Figure S2, respectively.

### Population accuracy

**FIGURE 7:**
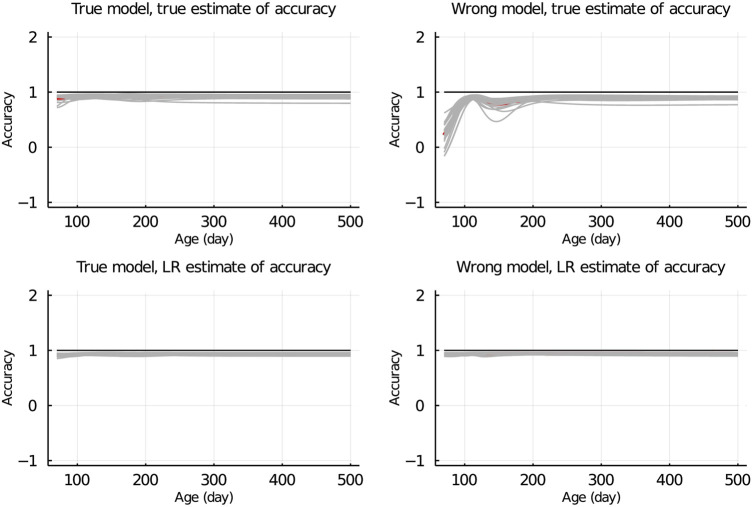
True and LR estimates of accuracy when the true or wrong model was fitted and when partitioning the data between animals. The true accuracy is defined as the correlation between true breeding values *u* and estimated breeding values of validation set based on partial set ${\hat{u}}_{p}$ . The LR estimates of accuracy is defined as $\frac{cov\left({\hat{u}}_{w},{\hat{u}}_{p}\right)}{\sqrt{\hat{var\left(u\right)}\times var\left({\hat{u}}_{p}\right)}}$ , where $\hat{var\left(u\right)}$ refers to an estimate of the genetic variance of individuals in the validation set. Grey lines are results of 20 simulation replicates, the red line is the mean of 20 replicates, and the black line indicates accuracy = 1.

**FIGURE 8:**
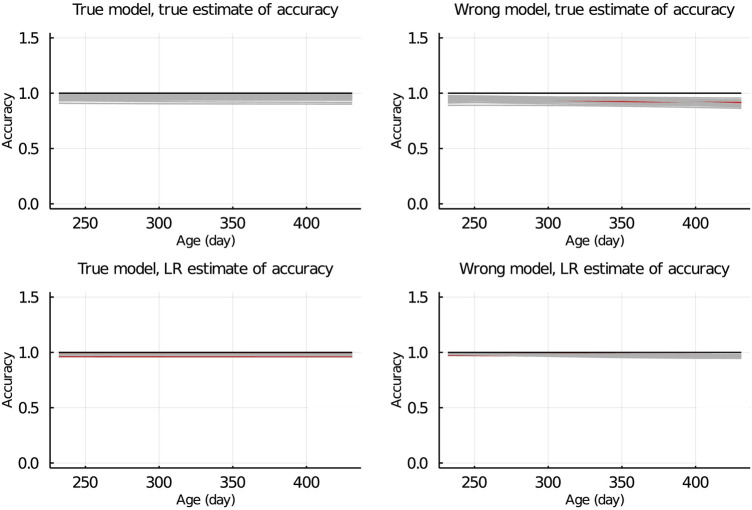
True and LR estimates of accuracy when the true or wrong model was fitted and when partitioning the data by age within animals. The true accuracy is defined as the correlation between true breeding values *u* and estimated breeding values of validation set based on partial set ${\hat{u}}_{p}$ . The LR estimates of accuracy is defined as $\frac{cov\left({\hat{u}}_{w},{\hat{u}}_{p}\right)}{\sqrt{\hat{var\left(u\right)}\times var\left({\hat{u}}_{p}\right)}}$ , where $\hat{var\left(u\right)}$ refers to an estimate of the genetic variance of individuals in the validation set. Grey lines are results of 20 simulation replicates, the red line is the mean of 20 replicates, and the black line indicates accuracy = 1.

In Figure 7, the true and LR estimates of prediction accuracy of EBV for body weights for each day when the data were partitioned between animals are presented. The LR estimates of prediction accuracy had a similar pattern as the true estimates of accuracy when using the true model but not when the wrong model was used. When partitioning the data by age within animals, the LR estimates of accuracy showed a similar pattern as the true estimates of accuracy, regardless of the model fitted (Figure 8). We also evaluated the difference between $cov\left(u,{\hat{u}}_{p}\right)$ and $cov\left({\hat{u}}_{w},{\hat{u}}_{p}\right)$ when fitting the true and wrong models for the three data partitioning scenarios (Table 1). There was a non-significant difference (*p* ≥ 0.74) between $cov\left(u,{\hat{u}}_{p}\right)$ and $cov\left({\hat{u}}_{w},{\hat{u}}_{p}\right)$ when the true model was fitted, but a significant difference (*p* ≤ 0.004) was observed for each scenario when the wrong model was fitted.

**TABLE 1:** Significance (*p*-values) of tests^1^ for the difference between $cov\left(u,{\hat{u}}_{p}\right)$ and $cov\left({\hat{u}}_{w},{\hat{u}}_{p}\right)$ for the three data partitioning scenarios and the two models.

	Between animals	By age within animals	Between animals and by age
True model	0.74	0.85	0.79
Wrong model	0.004	0.002	0.002

^1^H_0_: $cov\left(u,{\hat{u}}_{p}\right)=cov\left({\hat{u}}_{w},{\hat{u}}_{p}\right)$

## Discussion

Based on the initial idea by Reverter et al. (1994), Legarra and Reverter (2018) proposed the LR method to quantify the population accuracy of prediction of EBV implicitly assuming the fitted model is correct. They proved the validity of the LR method for EBV from a linear model using standard BLUP theory and applied the LR method to a real cattle data set (Legarra and Reverter, 2018). While the LR method has also been applied to EBV from a threshold model (Bermann et al., 2021), a mathematical proof of its validity for a non-linear method of prediction was not previously available. In this paper we provide the justification of the LR method for non-linear predictions.

The motivation for this paper came from a discussion on the validity of using the LR method to evaluate the prediction performance from a non-linear growth model, where marker effects are linked to body weight through latent variables. In this case, traditional CV cannot be used to obtain the accuracy of the latent variables because phenotypes are not available for these variables. However, in the LR method, $cov\left({\hat{u}}_{w},{\hat{u}}_{p}\right)$ is used to quantify $cov\left(u,{\hat{u}}_{p}\right)$ , assuming the fitted model is correct. So, the LR method can be applied to compute the accuracy for any random effect in the model. Predictions are also non-linear for analyses of threshold traits, survival traits, and whenever, in Bayesian analyses, parameters such as variance components are treated as unknowns with appropriate priors. The LR method also provides statistics to check whether the fitted model is adequate.

### Use of the LR method to estimate accuracy of prediction when the model is adequate

To generalize the LR method for linear or non-linear predictions, we presented a mathematical proof to justify using $cov\left({\hat{u}}_{w},{\hat{u}}_{p}\right)$ to quantify $cov\left(u,{\hat{u}}_{p}\right)$ even when the prediction is non-linear, provided that the fitted model is adequate. In our proof, we assumed that the partial data contains a subset of the phenotypes of the whole data. Belay et al. (2022) showed the LR method is also applicable to BLUP when the partial data contains a subset of the genotypes of the whole data. The proof presented in the current paper is similar in principle to that provided by Belay et al. (2022). Taken together, these two proofs show that the LR method is applicable to predictions based on the conditional mean, regardless of whether the data are partitioned by genotypes or phenotypes and regardless of whether the model is linear or non-linear.

### Use of the LR method to test model adequacy

We used simulated longitudinal data to investigate the ability of the LR method to determine whether the fitted model is adequate. In the context of our situation, the non-linear relationship is between the age of the animal and its weight, which makes how the data are partitioned into training and validation sets important. To explore how the strategy for partitioning the data into training and validation sets affects the ability of the LR method to determine whether the fitted model is adequate, three data partitioning strategies were used: between animals, by age within animals, and between animals and by age. We did not simulate selection in our data because we were comparing two models of growth rather than two models of inheritance. When the fitted model for growth is not adequate and the data are partitioned by age within animals, predictions from the training data would not be accurate even without selection. Thus, selection was not required to investigate properties of the LR method in this context. Further, in our analysis of the simulated data, the QTL were used as markers, for simplicity, but this does not influence the conclusions of our study either.

### Effect of data partitioning strategy on test of model adequacy

Below, we summarize the implications of the data partitioning strategies on the performance of the LR method, thereby providing guidelines for using the $\hat{\Delta }$ and ${\hat{b}}_{wp}$ to determine whether the fitted model is adequate. Significant deviations of the LR statistics from their expectations under the correct model were tested by replication of the simulations. In real data analyses, repeated k-fold LR can be used to test significance of model inadequacy using the two LR statistics. Alternatively, if a Bayesian method is employed for the LR analysis, the posterior probability of model inadequacy can be computed from a single partitioning of the data.

When the wrong model (BHQGM) was fitted and the data were partitioned between animals, the Δ was significant, but the $\hat{\Delta }$ was not able to detect this inadequate model (Figure 3). Macedo et al. (2020) also observed that for a certain misspecification of the model, the LR method was not able to correctly detect and estimate Δ. Figure 5 shows that when the wrong model was used, the true estimate of regression (the regression of *u* on ${\hat{u}}_{p}$ ) had a significant deviation from 1, and in this case the estimate of the regression coefficient based on the LR method was also significantly different from 1, although differing in magnitude from the true estimate of the regression coefficient. This is also consistent with the results observed by Macedo et al. (2020). The effect of fitting the true or the wrong model on the behavior of EBV against age for the partial and whole data were presented in Figure 2 (left column) for a randomly selected validation individual and data partitioning was by individual rather than age. In this case, when the wrong model was fitted, the EBV from the partial and whole data sets deviated more from the true BV than EBV from the true model did. However, even when the wrong model was used, the EBV from partial and whole data sets were very similar because the data were partitioned by individuals rather than age. This explains why the $\hat{\Delta }$ based on the LR method was not significant when the wrong model was used. This is because of when the data were partitioned between animals, there is no difference in the distribution of phenotypic values in training and validation sets, because we did not simulate selection and partitioning was not by generation. Thus the mean of ${\hat{u}}_{p}$ was not significantly different from mean of ${\hat{u}}_{w}$ .

Recall that in the LR method, the regression of *u* on ${\hat{u}}_{p}$ is estimated by the regression of ${\hat{u}}_{w}$ on ${\hat{u}}_{p}$ . In general, we expect that this statistic can be used to determine whether the model that is fitted is inadequate, in any study even when there is no difference in the distribution of phenotypic values in training and validation sets, such as when the data are partitioned by generation and there is no selection. The reason for this is that animals in the validation set will have a range of values for *u*, ${\hat{u}}_{p}$ , and ${\hat{u}}_{w}$ , and thus there is information to estimate the regression of *u* on ${\hat{u}}_{p}$ . In fact, the variance of *u* in the validation set can be even higher without selection compared to with selection, where for example, the validation animals are all from the last generation of selection. In the LR method, these values for ${\hat{u}}_{w}$ and ${\hat{u}}_{p}$ are used to estimate the regression of *u* on ${\hat{u}}_{p}$ . The variance of this regression estimator is proportional to $\frac{1}{Var\left({\hat{u}}_{p}\right)}$ , and thus, it is possible that this regression coefficient can be estimated even more accurately when there is no selection.

When the data were partitioned by age within animals, the LR method was able to correctly detect that the model was inadequate using $\hat{\Delta }$ and ${\hat{b}}_{wp}$ when the wrong model was used (Figures 4, 6). Figure 2 (middle column) shows the EBV of a randomly selected individual when the data were partitioned between animals. When the wrong model was fitted, the EBV estimated from partial and whole data sets both deviated from the true BV but the EBV based on the partial set was quite different from that estimated from the whole set. This illustrates the significant $\hat{\Delta }$ that was detected by the LR method for this scenario. Results for the partitioning between animal and by age (right column in Figure 2) were similar to those when partitioning by age within animals.

The inconsistency between $\hat{\Delta }$ and ${\hat{b}}_{wp}$ for different data partitioning strategies suggests that the LR method captures different aspects of the model for different data partitions. When the data were partitioned between animals, both the partial and whole data sets included phenotypes over the range from 70 to 500 days. Thus the fit of a phenotyped individual’s growth curve based on the partial and whole data sets were similar, even for the wrong model, although the fit might deviate from that using the true model. Using the wrong growth model only provides an incorrect fit to the relationship between age and body weight within individual but does not affect the flow of information between relatives. Thus to appropriately test the predictive ability of the fitted growth model using the LR method, we had to predict the body weights of animals that are outside the observed age range for animals in the training set, as was done when the data are partitioned by age. In this case, the partial data set has only body weights measured at ages up to 300 days, whereas the validation data set has body weights measured at ages up to 500 days. Thus when we predict the body weights in the validation set based on the fit of the growth model from the partial data set, we are testing the predictive ability of the growth model, rather than the predictive ability of data from older animals to predict younger animals. In this case, the LR method was able to correctly detect that the model was inadequate when the wrong model was used (bottom right plots in Figures 4, 6).

### Guidelines for data partitioning

In general, to properly test the model adequacy using the LR method, we need to use the model to predict the performance of individuals that have values for the relevant predictor variables or combination of predictor variables that were not present in the training data. In our simulated data, the predictor variables included the marker genotypes, as well as age. Let’s define the predicted performance of individual *i* as $\hat{y_{i}}=f\left(x_{i};\hat{\theta }\right)$ , where *f*(.) is the linear or non-linear function used for prediction, **x**_**i**_ is a vector of predictor variables for individual *i*, and $\hat{\theta }$ is the vector of estimates of model parameters. Below we will use genomic prediction by ridge regression BLUP (RR-BLUP) as an example for illustration. To evaluate the predictive ability of RR-BLUP, the data are partitioned into training and validation sets. The training set is used to fit the predictive model *f*(.) and to estimate the model parameters ***θ*** (i.e., marker effects). By plugging the marker effect estimates $\hat{\theta }$ and observed marker genotypes **x** into *f*(.), the performance of individuals in the validation set can be predicted. The predictive ability of the model is then quantified by comparing the predicted and observed performances of individuals in the validation set. In RR-BLUP, the relevant predictor variables are the marker genotypes. Thus the training and validation sets cannot include the same individual or individuals with the same genotypes across all loci. In our study, the LR method was used to determine whether it could detect an inadequate model when a wrong model was used for the growth curve, where the predictor is age and the dependent variable is BW (longitudinal BW). When the data were partitioned between animals, the training (partial) and validation sets included phenotypes for animals with age ranging from days 70–500, i.e., the same age range as used in the training data was used for the validation data and, therefore, $\hat{\Delta }$ failed to detect the use of an inadequate model. However, when the data were partitioned by age within animals, the model was trained using phenotypes with ages ranging from days 70–300 and it was tested by predicting body weights for animals with age ranging from days 301–500. In this case, the LR method was able to detect model inadequacy using $\hat{\Delta }$ . This was even true when the same genotypes were used in both the training and validation sets, because to check if the model used for predicting longitudinal BW is correct, the relevant predictor variable is age.

## Conclusion

We provide a mathematical proof for the validity of the LR method to estimate the accuracy of predictions based on the conditional mean, regardless of whether predictions are a linear or non-linear function of data, provided the fitted model is adequate. In the LR method, two statistics are used to check model adequacy. We found that the $\hat{\Delta }$ statistic can detect an inadequate model only when the data are partitioned such that the values of relevant predictor variables differ between the training and validation sets. On the other hand, the regression statistic was able to detect an inadequate model even when relevant predictor variables did not differ between the training and validation sets.

## Data Availability

The original contributions presented in the study are included in the article/Supplementary Material, further inquiries can be directed to the corresponding authors.

## Supplementary material

SUPPLEMENTARY FIGURE S1

$\Delta =\bar{{\hat{u}}_{p}}-\bar{u}$ and $\hat{\Delta }=\bar{{\hat{u}}_{p}}-\bar{{\hat{u}}_{w}}$ of EBV of body weights at each day when the true or wrong model was fitted and when partitioning the data between animals and by age. Grey lines are results of 20 simulation replicates, the red line is the mean of 20 replicates, and the black line indicates bias = 0. P refers to significance of tests for the difference between Δ or $\hat{\Delta }$ and 0.

SUPPLEMENTARY FIGURE S2 True and LR estimates of regression coefficient of EBV of body weights at each day when the true or wrong model was fitted and when partitioning the data between animals and by age. The true and LR estimates of regression coefficient are defined by regressing *u* on ${\hat{u}}_{p}$ , and ${\hat{u}}_{w}$ on ${\hat{u}}_{p}$ , respectively. Grey lines are results of 20 simulation replicates, the red line is the mean of 20 replicates, and the black line indicates regression coefficient = 1. P refers to significance of tests for the difference between true or LR estimate of regression coefficient and 1.

SUPPLEMENTARY FIGURE S3 True and LR estimates of accuracy of EBV of body weights at each day when the true or wrong model was fitted and when partitioning the data between animals and by age. The true accuracy is defined as the correlation between true breeding values *u* and estimated breeding values of validation set based on partial set ${\hat{u}}_{p}$ . The LR estimates of accuracy is defined as $\frac{cov\left({\hat{u}}_{w},{\hat{u}}_{p}\right)}{\sqrt{\hat{var\left(u\right)}\times var\left({\hat{u}}_{p}\right)}}$ ,where $\hat{var\left(u\right)}$ refers to an estimate of the genetic variance of individuals in the validation set. Grey lines are results of 20 simulation replicates, the red line is the mean of 20 replicates, and the black line indicates accuracy = 1.
