# Supplementary figures and images for: Use of the linear regression method to evaluate population accuracy of predictions from non-linear models
Source: Front Genet. 2024 May 31;15:1380643. doi: 10.3389/fgene.2024.1380643 (PMC11185077; doi:10.3389/fgene.2024.1380643)

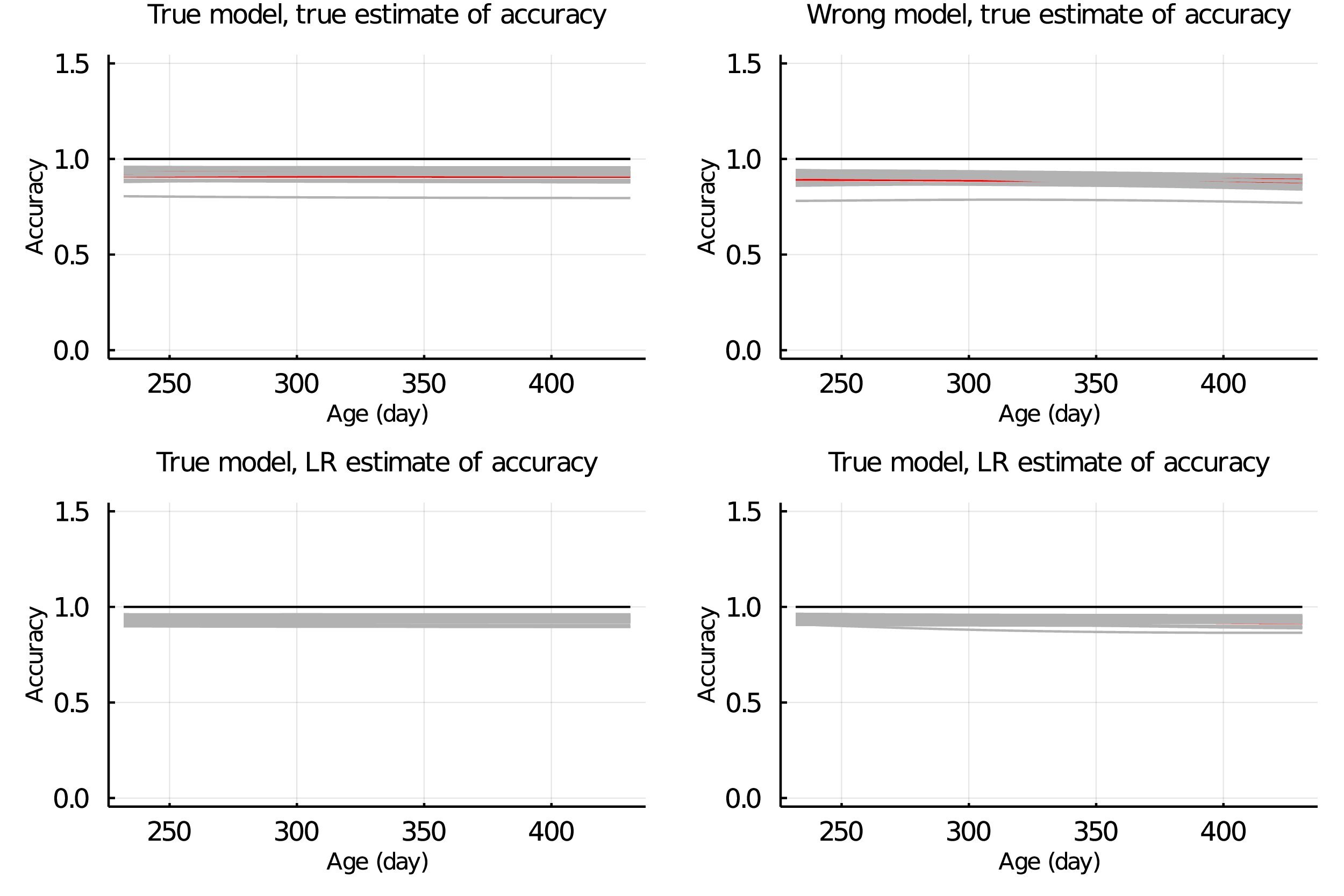

Supplement: Supplementary file 1 [file Image3.JPEG]

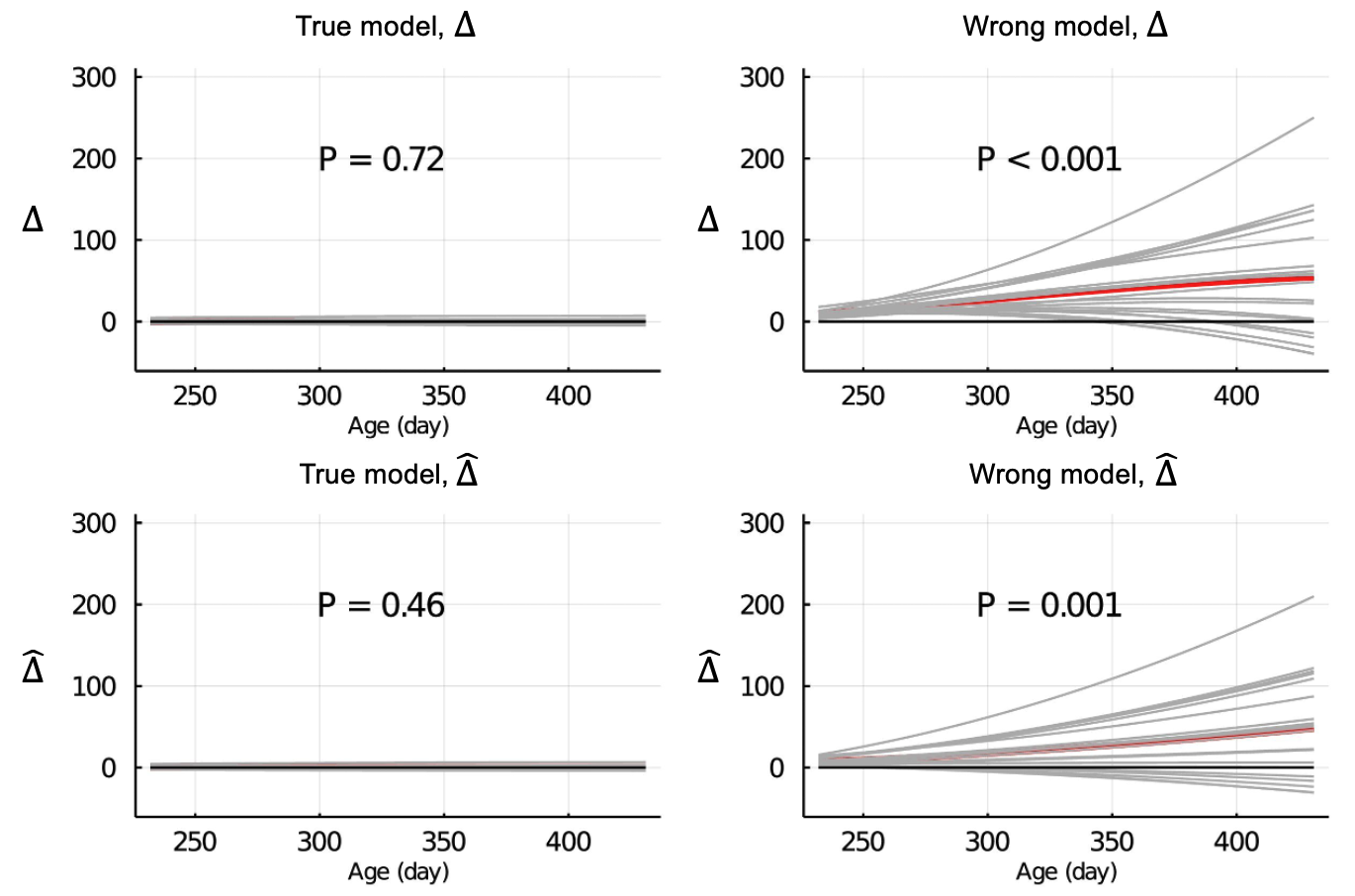

Supplement: Supplementary file 2 [file Image1.JPEG]

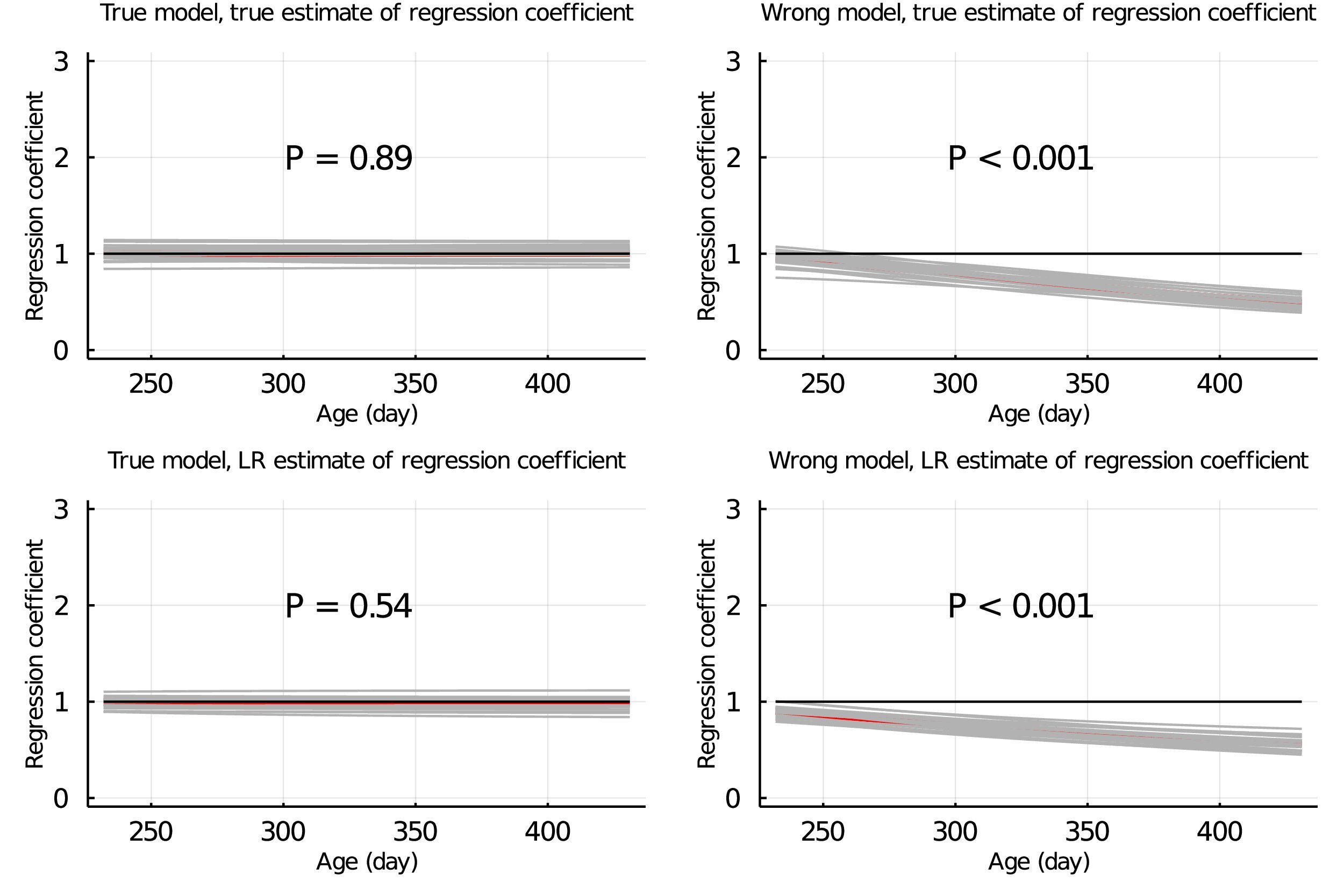

Supplement: Supplementary file 3 [file Image2.JPEG]
