# Supplementary material for: Use of the linear regression method to evaluate population accuracy of predictions from non-linear models
Source: Front Genet. 2024 May 31;15:1380643. doi: 10.3389/fgene.2024.1380643 (PMC11185077; doi:10.3389/fgene.2024.1380643)
Supplement: Supplementary file 4 [file DataSheet1.pdf]

Appendix

Proof of $E_{\mathbf{y}_r|\mathbf{y}_p}(\hat{\mathbf{u}}_w|\mathbf{y}_p) = \hat{\mathbf{u}}_p$

The estimated breeding values obtained from whole data ($\hat{\mathbf{u}}_w$) can be written as:

$$E_{\mathbf{y}_r|\mathbf{y}_p}(\hat{\mathbf{u}}_w|\mathbf{y}_p),$$

which is the expectation over the conditional distribution of $\mathbf{y}_r$ given $\mathbf{y}_p$. This can be explicitly rewritten as the following integral:

$$= \int_{\mathbf{y}_r} \hat{\mathbf{u}}_w \cdot f(\mathbf{y}_r|\mathbf{y}_p) d\mathbf{y}_r.$$

The conditional mean $\hat{\mathbf{u}}_w$ in the above expression is the inner integral over $\mathbf{u}$ in the expression given below:

$$= \int_{\mathbf{y}_r} \int_{\mathbf{u}} \mathbf{u} \cdot f(\mathbf{u}|\mathbf{y}_p, \mathbf{y}_r) d\mathbf{u} f(\mathbf{y}_r|\mathbf{y}_p) d\mathbf{y}_r.$$

In the above expression, substituting $f(\mathbf{u}|\mathbf{y}_p, \mathbf{y}_r)$ with $\frac{f(\mathbf{u}, \mathbf{y}_p, \mathbf{y}_r)}{f(\mathbf{y}_p, \mathbf{y}_r)}$, it can be rewritten as:

$$= \int_{\mathbf{y}_r} \int_{\mathbf{u}} \mathbf{u} \cdot \frac{f(\mathbf{u}, \mathbf{y}_p, \mathbf{y}_r)}{f(\mathbf{y}_p, \mathbf{y}_r)} d\mathbf{u} \cdot f(\mathbf{y}_r|\mathbf{y}_p) d\mathbf{y}_r,$$

where the $f(\mathbf{y}_p, \mathbf{y}_r)$ in the denominator can be written as a conditional distribution multiplied by a marginal distribution:

$$= \int_{\mathbf{y}_r} \int_{\mathbf{u}} \mathbf{u} \cdot \frac{f(\mathbf{u}, \mathbf{y}_p, \mathbf{y}_r)}{f(\mathbf{y}_r|\mathbf{y}_p) \cdot f(\mathbf{y}_p)} d\mathbf{u} \cdot f(\mathbf{y}_r|\mathbf{y}_p) d\mathbf{y}_r.$$

By interchanging the order of the integration in this double integral, we obtain:

$$= \int_{\mathbf{u}} \int_{\mathbf{y}_r} \mathbf{u} \cdot \frac{f(\mathbf{u}, \mathbf{y}_p, \mathbf{y}_r)}{f(\mathbf{y}_r|\mathbf{y}_p) \cdot f(\mathbf{y}_p)} \cdot f(\mathbf{y}_r|\mathbf{y}_p) d\mathbf{y}_r d\mathbf{u}.$$

Canceling $f(\mathbf{y}_r|\mathbf{y}_p)$ in the numerator and denominator of the expression above, it becomes:

$$= \int_{\mathbf{u}} \mathbf{u} \cdot \int_{\mathbf{y}_r} \frac{f(\mathbf{u}, \mathbf{y}_p, \mathbf{y}_r)}{f(\mathbf{y}_p)} d\mathbf{y}_r d\mathbf{u}.$$

Now, integrating over $\mathbf{y}_r$, we get:

$$= \int_{\mathbf{u}} \mathbf{u} \cdot \frac{f(\mathbf{u}, \mathbf{y}_p)}{f(\mathbf{y}_p)} d\mathbf{u},$$

which can be rewritten as:

$$= \int_{\mathbf{u}} \mathbf{u} \cdot f(\mathbf{u}|\mathbf{y}_p) d\mathbf{u}.$$

By integrating over $\mathbf{u}$, this becomes:

$$E(\mathbf{u}|\mathbf{y}_p) = \hat{\mathbf{u}}_p.$$

Proof of $E_{\mathbf{y}_w}[(\hat{\mathbf{u}}_w - \hat{\mathbf{u}}_p)(\hat{\mathbf{u}}_p - \boldsymbol{\theta}_p)'] = E_{\mathbf{y}_p}\{E_{\mathbf{y}_r|\mathbf{y}_p}[(\hat{\mathbf{u}}_w - \hat{\mathbf{u}}_p)(\hat{\mathbf{u}}_p - \boldsymbol{\theta}_p)'|\mathbf{y}_p]\}$

Without loss of generality, our proof is for a scalar function of $\mathbf{y}_p$ and $\mathbf{y}_r$ rather than for a matrix. Suppose, we are interested in computing the expected value of some scalar function $g(\mathbf{y}_r, \mathbf{y}_p)$. We show below that $E_{\mathbf{y}_r, \mathbf{y}_p}[g(\mathbf{y}_r, \mathbf{y}_p)] = E_{\mathbf{y}_p}\{E_{\mathbf{y}_r|\mathbf{y}_p}[g(\mathbf{y}_r, \mathbf{y}_p)|\mathbf{y}_p]\}$, which can be extended to prove $E_{\mathbf{y}_w}[(\hat{\mathbf{u}}_w - \hat{\mathbf{u}}_p)(\hat{\mathbf{u}}_p - \boldsymbol{\theta}_p)'] = E_{\mathbf{y}_p}\{E_{\mathbf{y}_r|\mathbf{y}_p}[(\hat{\mathbf{u}}_w - \hat{\mathbf{u}}_p)(\hat{\mathbf{u}}_p - \boldsymbol{\theta}_p)'|\mathbf{y}_p]$. The right-hand side of this expression can be written explicitly as the following integral:

$$E_{\mathbf{y}_p}\{E_{\mathbf{y}_r|\mathbf{y}_p}[g(\mathbf{y}_r, \mathbf{y}_p)|\mathbf{y}_p] = \int_{\mathbf{y}_p} \left[\int_{\mathbf{y}_r} g(\mathbf{y}_r, \mathbf{y}_p) \cdot f(\mathbf{y}_r|\mathbf{y}_p) d_{\mathbf{y}_r} \right] \cdot f(\mathbf{y}_p) d_{\mathbf{y}_p},$$

where the inner expectation on the left-hand side is the expected value of $g(\mathbf{y}_r, \mathbf{y}_p)$ over the conditional distribution of $\mathbf{y}_r$ given $\mathbf{y}_p$. Now, by recognizing that $f(\mathbf{y}_r|\mathbf{y}_p) \cdot f(\mathbf{y}_p)$ equals to the joint distribution of $\mathbf{y}_r$ and $\mathbf{y}_p$, the right-hand side of the above expression becomes

$$\int_{\mathbf{y}_p} \left[\int_{\mathbf{y}_r} g(\mathbf{y}_r, \mathbf{y}_p) \cdot f(\mathbf{y}_r|\mathbf{y}_p) \cdot f(\mathbf{y}_p) d_{\mathbf{y}_r} \right] d_{\mathbf{y}_p}$$

$$= \int_{\mathbf{y}_p} \int_{\mathbf{y}_r} g(\mathbf{y}_r, \mathbf{y}_p) \cdot f(\mathbf{y}_r, \mathbf{y}_p) d_{\mathbf{y}_r} d_{\mathbf{y}_p}$$

$$= E_{\mathbf{y}_r, \mathbf{y}_p}[g(\mathbf{y}_r, \mathbf{y}_p)].$$

This is a commonly used strategy for computing expectations of random functions (Rao, 1973).

References

Rao, C. R. (1973). *Linear statistical inference and its applications*, volume 2. Wiley New York.
